# Cardiac Contractility Modulation as bridge to recovery in a patient with advanced heart failure evaluated for left ventricular assist devices: a case report and review of literature

**DOI:** 10.3389/fcvm.2025.1516574

**Published:** 2025-07-03

**Authors:** Francesca Menichetti, Marco Solari, Elisa Salvetti, Vincenzo Guarnaccia, Tommaso Girasole, Flavio Venturi, Attilio Del Rosso

**Affiliations:** Cardiology Unit, Department of Internal Medicine, San Giuseppe Hospital, Empoli, Italy

**Keywords:** Cardiac Contractility Modulation (CCM), advance heart failure, bridge to recovery, left ventricular assist devices (LVAD), heart transplantation

## Abstract

This case report illustrates the effects of CCM as a bridge to recovery in a patient who has been a candidate for LVAD. After CCM the patient hase become stable in NYHA class II, with no further acute HF episodes and a significant clinical and functional improvements. Consequently, the patient was no longer an LVAD candidate, as CCM had acted as a bridge to recovery, making the advanced HF designation no longer applicable. The ease of implantation, uneventful procedural recovery, extraordinary device longevity, and favorable risk profile all position CCM as an important tool in the treatment of advanced HFrEF patients candidate to LVAD therapy, serving as a bridge to recovery.Further large-scale randomized controlled trials are needed to confirm the long-term benefits of CCM therapy in this particular population subgroup, helping a better patient selection.

## Introduction

Up to 10% of patients with heart failure (HF) fail to respond to pharmacological treatments, leading to a clinical progression (1). The preferred therapeutic approach for patients with advanced HF is heart transplantation (HT) or alternatively the implantation of a left ventricular assist device (LVAD) (2). Cardiac Contractility Modulation (CCM) represents an emerging therapeutic strategy for patients with heart failure with reduced ejection fraction (HFrEF) and could benefit this specific population candidate to HT or LVAD.

The CCM therapy affects the epigenetic and proteomic scenery of cardiomyocytes, by applying non-excitatory electrical signals to the myocardium during the absolute refractory period of the action potential. The mechanisms underlying CCM's effects remain unclear but may partly involve improved calcium handling through upregulation of L-type Ca2+ channels and regulation of phospholamban activity (3); this leads to enhancing contraction without increasing oxygen consumption. The final result is an improvement of exercise tolerance and quality of life and a reduction of hospital admissions for patient with HF and CCM device implanted.

The FIX-HF-5C2 trial evaluated 60 patients with an ejection fraction (EF) between 25% and 45%, NYHA class III-IV symptoms despite optimal medical therapy, and a narrow QRS interval (<130 ms) without indication for cardiac resynchronization therapy (CRT). After six months of follow-up, CCM demonstrated a reduction in HF-related hospitalizations and an improvement in exercise capacity, quality of life and NYHA class (4).

Similarly, the CCM-REG registry analyzed outcomes of 140 patients with an EF between 25% and 45%, already implanted with CCM devices. The primary endpoint was a comparison between predicted survival by the Seattle Heart Failure Model (SHFM) to that observed through a follow-up of 3 years. While no significant difference in survival was observed both in the entire cohort and in the one with LVEF between 25% and 34%, patients with an EF between 35% and 45% exhibited a better survival compared to SHFM predictions (5).

Another registry, which included 68 NYHA class II–III patients with low EF and a QRS duration <130 ms, reported a significant reduction in mortality over a follow-up period of 4.5 years compared to SHFM-predicted mortality (6). This better survival was also observed in other registries (7, 8).

A recent case report suggested that CCM may be a viable bridge-to-transplant strategy in select end-stage HFrEF patients not adequately compensated by pharmacological therapy in which LVAD was contraindicated due to factors such as severe obesity (9).

Additionally, the management of systolic HF post-HT remains challenging due to drug interactions and renal insufficiency with subsequent limitations to optimal medical therapy. A case report documented the first use of CCM in a post-HT patient with refractory HF and narrow QRS (10).

This case report illustrates the effects of CCM as a bridge to recovery in a patient who was a candidate for LVAD. This clinical outcome may influence the approach for treating many patients with advanced-stage heart failure in any therapeutic phases.

## Case presentation

We describe the case of a 74-year-old patient presenting to our Cardiology Department with worsening dyspnea at rest. The patient's medical history included dilated cardiomyopathy with severe left ventricular dysfunction and a dual-chamber ICD implanted for primary prevention 5 years before; ICD memory revealed an episode of appropriate therapy delivered by the ICD for ventricular fibrillation 2 years before. Despite receiving optimal medical therapy at the highest tolerated doses (sacubitril/valsartan 49/51 mg twice daily, bisoprolol 5 mg daily, and eplerenone 50 mg daily), severe left ventricular dysfunction persisted and the patient continued to experience NYHA class III symptoms. In the last 12 months, the patient was hospitalized several times due to HF recurrences, requiring increased home diuretic therapy. At an outpatient visit, we used echocardiographic speckle-tracking strain imaging (a non-Doppler and angle-independent technique) for myocardial function assessment (11). This revealed an impaired global longitudinal strain (GLS −10.0%).

A cardiopulmonary testing confirmed severe functional impairment, with a peak oxygen uptake (VO2 peak) of 11 ml/kg/min and a ventilation/carbon dioxide production slope (VE/VCO2) of 36. After few weeks the patient was admitted for cardiogenic shock (CS). A severely depressed systolic function (LVEF 17% calculated with the biplane Simpson's formula) and widespread hypokinesia of the LV were documented. Right ventricular function was also severely impaired. The patient exhibited systolic blood pressure <90 mmHg for ≥30 min, refractory to fluid resuscitation, with laboratory findings consistent with end-organ dysfunction. The cardiac index (CI), assessed via FloTrac/Vigileo (12), was 1.9 L/min/m^2^.

Given the marked functional impairment despite optimized therapy, the patient was evaluated for HT but excluded for age limits. Few week after hospital discharge, despite ambulatory pulsed levosimendan infusion, the patient experienced another episode of acute HF, requiring hospitalization and continuous inotropic support. The patient was subsequently evaluated for LVAD implantation. The efficacy of assist devices has been demonstrated in a randomized trial enrolling end-stage HF patients awaiting CT, with a significant survival and quality of life improvement by the use of an LVAD. However, only a quarter of patients survived to 2 years and complications of LVAD were frequent (13). Although fully magnetically levitated (centrifugal-flow LVADs are associated with better outcomes than axial-flow devices), infections related to the device frequently occur, limiting the actual indication (14). The patient's INTERMACS score (a commonly used measure of disease severity) at that time was 3 (stable continuous inotrope-dependent).

Considering the patient's small size, impaired right ventricular function and high risk of infection, device malfunction and pump thrombosis associated with LVAD in patients with pre-existing cardiac devices, CCM implantation was identified as the most feasible therapeutic alternative. Patient has been informed about the benefits, expectations, and potential complications of CCM implantation, both in writing and verbally.

**Figure 1:**
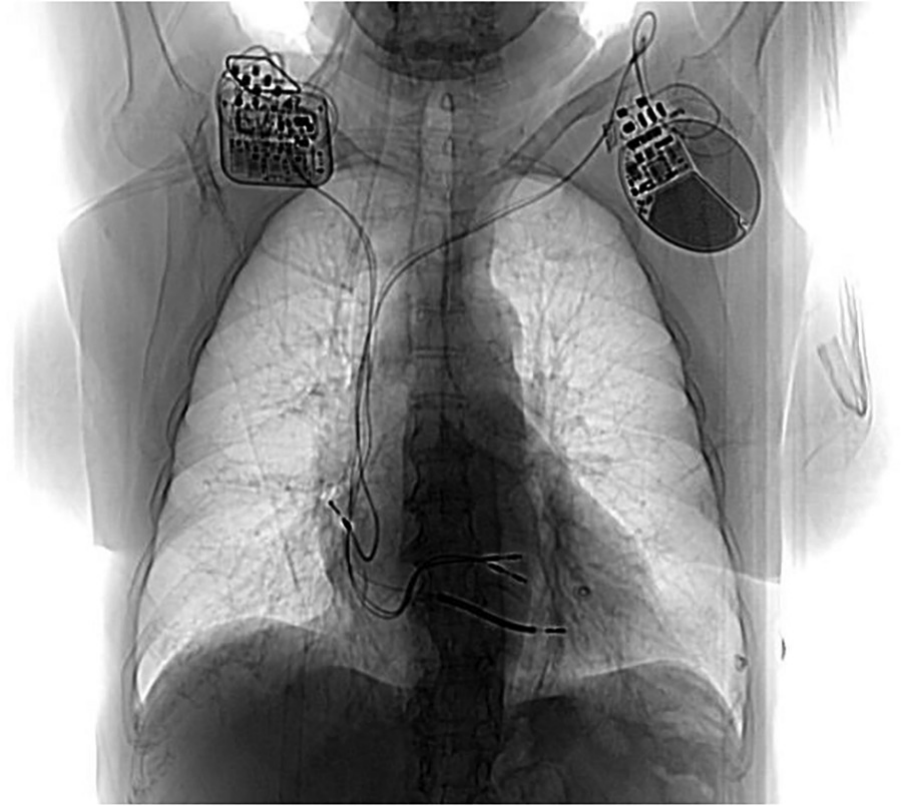
Post CCM implant chest X ray. Generator is located in the right infraclavicular fossa and it’s connected to 2 ventricular pacing leads. On the left side the previous dual chamber ICD can be noted.

**Figure 2:**
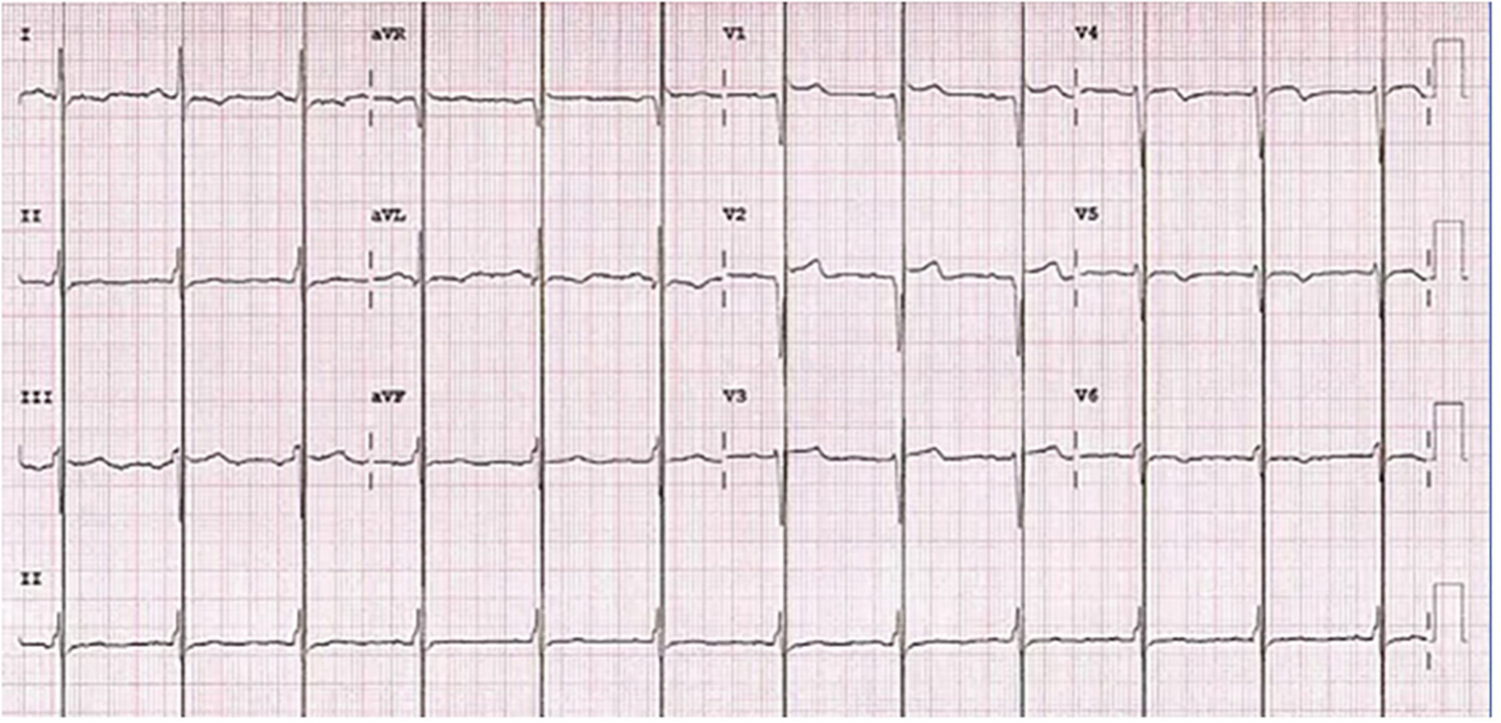
Electrocardiogram during cardiac contractility modulation treatment with typical spike within QRS refractory period.

Following informed consent on the benefits, expectations, and potential complications of CCM implantation, in December 2022 the patient underwent implantation of a two-lead CCM, consisting of an Optimizer Smart implantable pulse generator (Impulse Dynamics Inc. Orangeburg, NY, USA) located in the right infraclavicular fossa connected to 2 ventricular pacing leads placed in the interventricular septum (Figure 1). The target area is the lower part of the septal region of the right ventricle, maintaining at least 2 cm separation. Lead positioning is obtained with a Mond type stylet, designed with a primary J curve and a secondary posterior curve. Proper pacing and sensing parameters were confirmed, with no diaphragmatic or chest wall capture and no patient's complain of chest discomfort during high-output stimulation. The device was programmed in OVO-LS-CCM mode with a CCM train of 2 pulses of 7.5 V with a 22 ms duration and a CCM programmed dose of 8 h periods per day. The typical CCM ECG is with spike within QRS refractory period (Figure 2).

**Figure 3:**
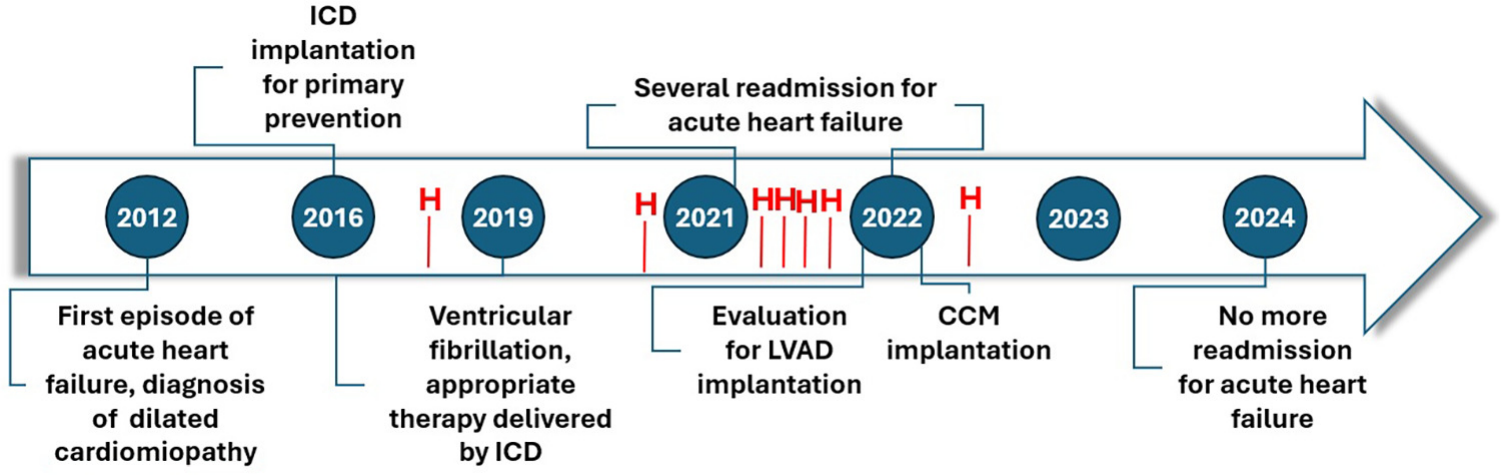
Timeline of patient’s history. No further hospitalization (H) were observed for acute HF episodes after three months by CCM implant.

A innovating feature of the Optimizer Smart device is its rechargeable battery requiring the patient to charge it with a portable device for only 1 h per week. This rechargeable battery is guaranteed for 15 years of longevity. The patient has been educated about the importance of battery charge and the knowledge of appropriate actions to take in response to device alarms.

In the first two months post-implantation, no symptom improvement was observed and the patient required another hospitalization for acute decompensated heart failure. However, by the third month, gradual symptomatic improvement was observed, including enhanced NYHA class, six-minute walk test distance, echocardiographic parameters, and NT-proBNP levels. The LV parameters also improved, with GLS reaching −42% (vs. −10%) and LVEF 35% (vs. 17%). Six months post-implantation walked 350 meters without desaturation at SMWT the patient and her MLWHFQ score improved to 51. A repeat cardiopulmonary test showed a VO2 peak increase to 15.6 ml/kg/min (vs. 11 ml/kg/min) and a reduced VE/VCO2 slope of 22 (vs. 36). Since low ventilatory efficiency is an important predictor of cardiovascular mortality in HF patients (15), these results highlight CCM's therapeutic impact on our patient. By December 2024, the patient was stable in NYHA class II, with no further acute HF episodes (Figure 3). The patient's INTERMACS score was unquantifiable due to significant clinical and functional improvements. Consequently, the patient was no longer an LVAD candidate, as CCM had acted as a bridge to recovery, making the advanced HF designation no longer applicable. Finally, the patient perspective has been a new restart provided by CCM.

## Discussion

The management of advanced HF presents significant challenges. In patients without CRT indications, HT remains the gold standard but is limited by donor shortages, while LVAD therapy is often limited by HF patient comorbidities. The CCM therapy aims to enhance myocardial contractility and could been chosen as additional therapy for patients with severe HF.

Currently, no large-scale randomized trials have demonstrated mortality endpoints with CCM, so that therapy has not yet been implemented in the guidelines. However, the evidence supporting CCM is comparable with the one supporting the CRT, indeed both these therapies have not been yet showed benefit in survival endpoint. On the other hand, meta-analyses and clinical trials have shown significant improvements in cardiopulmonary function, suggesting it as a viable option for CRT-non-responsive patients with a wide QRS duration and patients with reduced LVEF and “narrow” QRS (16–18). Notably, EF inclusion criteria (EF 25%–45%) for CCM encompass twice as many patients as are currently indicated for CRT. Furthermore, over 80% of CCM patients improve by at least one NYHA class and over 40% of CCM patients improve two classes. Although tricuspid regurgitation is a potential complication after the implantation of right ventricular electrodes, preliminary data suggest no significant worsening with CCM therapy (19). Finally recent advancements have led to the first successful implantations of devices integrating ICD and CCM functionalities (20). This new development is a promising area for ongoing research and may further expand the role of CCM.

Subgroup analysis from the FIX-HF-5 trial indicated limited benefit in patients with EF <25% (21); however, few effective therapies exist for this kind of patients and no worsening was observed in this group. Our patient belongs to this population subgroup with severely reduced LVEF and advanced heart failure so LVAD could have served as a bridge to HT and CCM does not seem to promise great benefit. However, CCM was chosen as an alternative therapeutic option and after its implantation the patient returned to NYHA class II, regained a quality of life in many daily activities and experienced significant improvements in echocardiographic and cardiopulmonary test data. Consequently, the patient was no longer an LVAD candidate, as CCM had acted as a bridge to recovery.

Nowadays a prospective worldwide registry is needed, with strong monitoring in specific populations and avoiding excessive reliance on mortality endpoints that could miss the real clinical benefits of CCM therapy.

## Conclusion

This case highlights CCM therapy as a bridge to recovery in an advanced HFrEF patient initially considered for LVAD. The CCM therapy offers ease of implantation, minimal procedural risks, long device longevity, and a favorable safety profile, serving as a bridge to recovery in selected end-stage HFrEF patients. Further randomized controlled trials are necessary to establish the long-term benefits of CCM therapy and refine patient selection criteria.

## Data Availability

The raw data supporting the conclusions of this article will be made available by the authors, without undue reservation.
